# Coronary events in elderly patients with non-valvular atrial fibrillation: a prespecified sub-analysis of the ANAFIE registry

**DOI:** 10.1007/s12928-024-00984-9

**Published:** 2024-02-13

**Authors:** Masato Nakamura, Hiroshi Inoue, Takeshi Yamashita, Masaharu Akao, Hirotsugu Atarashi, Takanori Ikeda, Yukihiro Koretsune, Ken Okumura, Wataru Shimizu, Shinya Suzuki, Hiroyuki Tsutsui, Kazunori Toyoda, Masahiro Yasaka, Takenori Yamaguchi, Satoshi Teramukai, Yoshiyuki Morishima, Masayuki Fukuzawa, Atsushi Takita, Atsushi Hirayama

**Affiliations:** 1 Division of Minimally Invasive Treatment in Cardiovascular Medicine, Toho University Ohashi Medical Center, 2-22-36, Ohashi, Meguro-Ku, Tokyo, 153-8515 Japan; 2 Saiseikai Toyama Hospital, Toyama, Japan; 3 The Cardiovascular Institute, Tokyo, Japan; 4 Department of Cardiology, National Hospital Organization Kyoto Medical Center, Kyoto, Japan; 5 AOI Hachioji Hospital, Tokyo, Japan; 6 Department of Cardiovascular Medicine, Toho University Faculty of Medicine, Tokyo, Japan; 7 National Hospital Organization Osaka National Hospital, Osaka, Japan; 8 Division of Cardiology, Saiseikai Kumamoto Hospital Cardiovascular Center, Kumamoto, Japan; 9 Department of Cardiovascular Medicine, Nippon Medical School, Tama Nagayama Hospital, Tokyo, Japan; 10 Department of Cardiovascular Medicine, Faculty of Medical Sciences, Kyushu University, Fukuoka, Japan; 11 Department of Cerebrovascular Medicine, National Cerebral and Cardiovascular Center, Osaka, Japan; 12 Department of Cerebrovascular Medicine, Fukuoka Neurosurgical Hospital, Fukuoka, Japan; 13 Department of Biostatistics, Graduate School of Medical Science, Kyoto Prefectural University of Medicine, Kyoto, Japan; 14 Primary Medical Science Department, Daiichi Sankyo Co., Ltd, Tokyo, Japan; 15 Data Intelligence Department, Daiichi Sankyo Co., Ltd., Tokyo, Japan; 16 Department of Medicine, Osaka Fukujuji Hospital, Neyagawa, Japan

**Keywords:** Coronary events, Elderly, Non-valvular atrial fibrillation, Coronary artery disease, Oral anticoagulant

## Abstract

Real-world data on coronary events (CE) in elderly patients with atrial fibrillation (AF) are lacking in the direct oral anticoagulant era. This prespecified sub-analysis of the ANAFIE Registry, a prospective observational study in > 30,000 Japanese patients aged ≥ 75 years with non-valvular AF (NVAF), investigated CE incidence and risk factors. The incidence and risk factors for new-onset CE (a composite of myocardial infarction [MI] and cardiac intervention for coronary heart diseases other than MI), MI, and cardiac intervention for coronary heart diseases other than MI during the 2-year follow-up were assessed. Bleeding events in CE patients were also examined. Among 32,275 patients, the incidence rate per 100 patient-years was 0.48 (95% confidence interval (CI): 0.42–0.53) for CE during the 2-year follow-up, 0.20 (0.16–0.23) for MI, and 0.29 (0.25–0.33) for cardiac intervention for coronary heart diseases other than MI; that of stroke/systemic embolism was 1.62 (1.52–1.73). Patients with CE (n = 287) likely had lower creatinine clearance (CrCL) and higher CHADS_2_ and HAS-BLED scores than patients without CE (n = 31,988). Significant risk factors associated with new-onset CE were male sex, systolic blood pressure of ≥ 130 mmHg, diabetes mellitus (glycated hemoglobin ≥ 6.0%), CE history, antiplatelet agent use, and CrCL < 50 mL/min. Major bleeding incidence was significantly higher in patients with new-onset CE vs without CE (odds ratio [95% CI], 3.35 [2.06–5.43]). In elderly patients with NVAF, CE incidence was lower than stroke/systemic embolism incidence. New-onset CE (vs no CE) was associated with a higher incidence of major bleeding.

*Trial registration*: UMIN000024006.

**Graphical Abstract:**

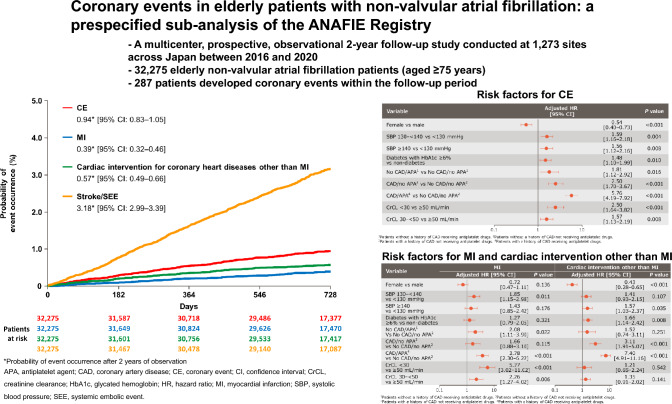

## Introduction

Atrial fibrillation (AF) is a major risk factor for stroke [1]. This is particularly relevant for elderly patients as the prevalence of AF increases with advancing age from 0.12–0.16% in people aged < 49 years to 1.7–4.0% among those aged 60–70 years; proportions may be as high as 13.5–17.8% among those aged > 80 years [2–4]. Both AF and coronary artery disease (CAD) are common cardiovascular conditions encountered in daily clinical practice in elderly patients. The diseases tend to coexist because of shared risk factors, such as hypertension, diabetes, advanced age, obesity, and smoking, and they have similar pathophysiological features, such as inflammation [5]. CAD is more common in patients with AF, ranging between 17% and 46.5% [6, 7]. Furthermore, several studies have reported that comorbid CAD and AF aggravate one another [8, 9]. AF is associated with a twofold increase in the risk of myocardial infarction (MI) [7]. According to the Framingham study, patients with AF and heart disease have a 2.2-times higher probability of developing new coronary events (CE) compared with patients with heart disease without AF [9]. CAD is the third leading cause of death worldwide, with approximately 18 million deaths annually attributed to CAD [10].

Patients with both CAD and AF pose additional challenges in terms of treatment—which includes rhythm management, anticoagulants, and antiplatelet agents (APA)—and may require more complex treatment strategies to mitigate possible increases in bleeding risk [5, 11]. The Japanese open-label AFIRE trial suggested that oral anticoagulant (OAC) monotherapy was superior for safety compared with OAC and APA combination therapy in patients with AF and CAD [12]. Additionally, a temporal association between major bleeding and subsequent cardiovascular events and death in patients with AF and stable CAD has been demonstrated [13].

Although clinical outcomes in AF patients based on the presence or absence of CAD (e.g., MI or percutaneous coronary intervention [PCI]) have been reported [10, 14–18], real-world data on the incidence and risk factors of CE in elderly patients—who are at the highest risk of events—are lacking, especially in the era of direct OACs (DOACs). The All Nippon Atrial Fibrillation In the Elderly (ANAFIE) Registry aimed to clarify the real-world clinical status and prognosis of elderly patients with non-valvular AF (NVAF) in Japan. Over 30,000 elderly (≥ 75 years of age) Japanese patients with NVAF were enrolled and followed up for 2 years to investigate anticoagulation therapy status and outcomes in routine clinical practice. The 2-year follow-up data [19] and several sub-analyses have been published [20–24].

The main objective of this prespecified sub-analysis of the ANAFIE Registry was to investigate the incidence and risk factors of CE in elderly Japanese patients with NVAF. The occurrence of bleeding events in CE patients was also examined.

## Methods

### Study design

The ANAFIE Registry was a multicenter, prospective, observational study conducted at 1273 sites across Japan between 2016 and 2020. Details of the study design and rationale have been published [25]. The trial was registered in the UMIN Clinical Trials Registry under the identifier UMIN000024006. The study was compliant with the Declaration of Helsinki and local requirements for registries. Ethics committees approved the study protocol. Written informed consent was obtained from patients or family members in case of communication disorders (i.e., aphasia) or cognitive impairment.

### Patients

Enrolled outpatients were men and women ≥ 75 years of age, diagnosed with NVAF by electrocardiography, who were able to attend hospital visits. Patients were excluded from enrollment if they were participating/planning to participate in an interventional study; had a definite diagnosis of mitral stenosis or artificial heart valve replacement (either mechanical or tissue valve prostheses), or had experienced very recent cardiovascular events, including stroke, MI, cardiac intervention, heart failure requiring hospitalization, or any bleeding leading to hospitalization within 1 month prior to enrollment; life expectancy of < 1 year; or who were deemed inappropriate for participation by treating physicians.

### Study endpoints

Specifically, in this pre-specified sub-analysis, we assessed the incidence for new-onset CE (defined as a composite of MI and cardiac intervention for coronary heart diseases other than MI), MI, cardiac intervention for coronary heart diseases other than MI, major bleeding, clinically relevant non-major bleeding (CRNMB), intracranial hemorrhage (ICH), and gastrointestinal (GI) bleeding during the 2-year follow-up period. Major bleeding was classified using the International Society on Thrombosis and Haemostasis definition.

### Statistical analysis

The Kaplan–Meier method was used to estimate the probability of occurrence of CE and other clinical events. The incidences of CE and other clinical events were also estimated as incidence rates per 100 person-years with 95% confidence intervals (CIs). A multivariate analysis was performed to identify risk factors of CE calculated using the Cox proportional hazards model. This analysis was also performed by combining the history of CAD and APA use. For bleeding events, based on the presence or absence of CAD, odds ratios (ORs) were evaluated using a logistic regression model adjusted for prognostic factors. Statistical tests were two-sided, with a significance level of 5%. The statistical software used for these analyses was SAS version 9.4 (SAS Institute, Tokyo, Japan).

## Results

### Patient disposition and characteristics

Of the 32,275 patients analyzed in the ANAFIE Registry, 287 developed CE (MI, cardiac intervention for coronary heart diseases other than MI) (0.89%). Table 1 shows the characteristics of patients with new-onset CE and those without CE. Significantly more men than women had CE vs no CE. Creatinine clearance (CrCL) was significantly lower, and CHADS_2_ and HAS-BLED scores were significantly higher in patients with CE vs those without CE. Similarly, significantly higher proportions of patients with CE had diabetes mellitus, dyslipidemia, a history of CAD including prior MI and/or angina, a history of cerebrovascular diseases including lacunar infarction and peripheral arterial disease and falls within 1 year.

**Table 1:** Background characteristics of patients at baseline by the presence of new-onset coronary events

	Total N = 32,275	CE n = 287	No CE n = 31,988	*P* value^a^
Male	18,482 (57.3)	210 (73.2)	18,272 (57.1)	< 0.001
Age, years	81.5 ± 4.8	81.2 ± 4.5	81.5 ± 4.8	0.438
Body mass index, kg/m^2^	23.3 ± 3.6	23.4 ± 3.3	23.3 ± 3.6	0.875
SBP, mmHg	127.4 ± 17.0	128.5 ± 17.8	127.3 ± 17.0	0.267
DBP, mmHg	70.6 ± 11.6	69.3 ± 11.5	70.7 ± 11.6	0.056
Creatinine clearance, mL/min	48.4 ± 18.2	44.1 ± 16.6	48.4 ± 18.2	< 0.001
CHADS_2_ score	2.9 ± 1.2	3.3 ± 1.3	2.9 ± 1.2	< 0.001
HAS-BLED score	1.9 ± 0.9	2.2 ± 1.0	1.9 ± 0.9	< 0.001
History of major bleeding	1439 (4.5)	14 (4.9)	1425 (4.5)	0.729
AF type				
Paroxysmal	13,586 (42.1)	129 (44.9)	13,457 (42.1)	0.397
Persistent	9701 (30.1)	76 (26.5)	9625 (30.1)	–
Permanent	8988 (27.8)	82 (28.6)	8906 (27.8)	–
OACs	29,830 (92.4)	265 (92.3)	29,565 (92.4)	0.954
Warfarin	8233 (25.5)	81 (28.2)	8152 (25.5)	0.278
DOACs	21,585 (66.9)	184 (64.1)	21,401 (66.9)	0.285
History of non-pharmacological therapy for AF	5677 (17.6)	56 (19.5)	5621 (17.6)	0.390
Catheter ablation	2970 (9.2)	24 (8.4)	2946 (9.2)	0.621
Comorbidities				
Hypertension	24,312 (75.3)	228 (79.4)	24,084 (75.3)	0.104
Diabetes mellitus	8733 (27.1)	120 (41.8)	8613 (26.9)	< 0.001
Dyslipidemia	13,728 (42.5)	156 (54.4)	13,572 (42.4)	< 0.001
Chronic kidney disease	6705 (20.8)	66 (23.0)	6639 (20.8)	0.351
Myocardial infarction	1851 (5.7)	52 (18.1)	1799 (5.6)	< 0.001
Angina	5521 (17.1)	117 (40.8)	5404 (16.9)	< 0.001
Heart failure, left ventricular systolic dysfunction	12,116 (37.5)	123 (42.9)	12,154 (38.0)	0.091
Cerebrovascular disease	7303 (22.6)	85 (29.6)	7218 (22.6)	0.005
Atherosclerotic infarction	655 (2.0)	8 (2.8)	647 (2.0)	0.360
Cardiogenic infarction	2377 (7.4)	23 (8.0)	2354 (7.4)	0.672
Lacunar infarction	1436 (4.4)	20 (7.0)	1416 (4.4)	0.038
Peripheral arterial disease^b^	1931 (6.0)	41 (14.3)	1890 (5.9)	< 0.001
Fall within 1 year	2347 (7.3)	30 (10.5)	2317 (7.2)	0.026
Antiplatelet agents	5704 (17.7)	121 (42.1)	5583 (17.5)	< 0.001

Data are presented as *n* (%) or mean ± standard deviation

*AF* atrial fibrillation; *CE* coronary event; *DBP* diastolic blood pressure; *DOAC* direct oral anticoagulant; *OAC* oral anticoagulant; *SBP* systolic blood pressure

^a^Comparison between CE and No CE groups

^b^Aortic plaque, internal carotid artery stenosis, and arteriosclerosis obliterans

### Incidence of events

**Fig. 1:**
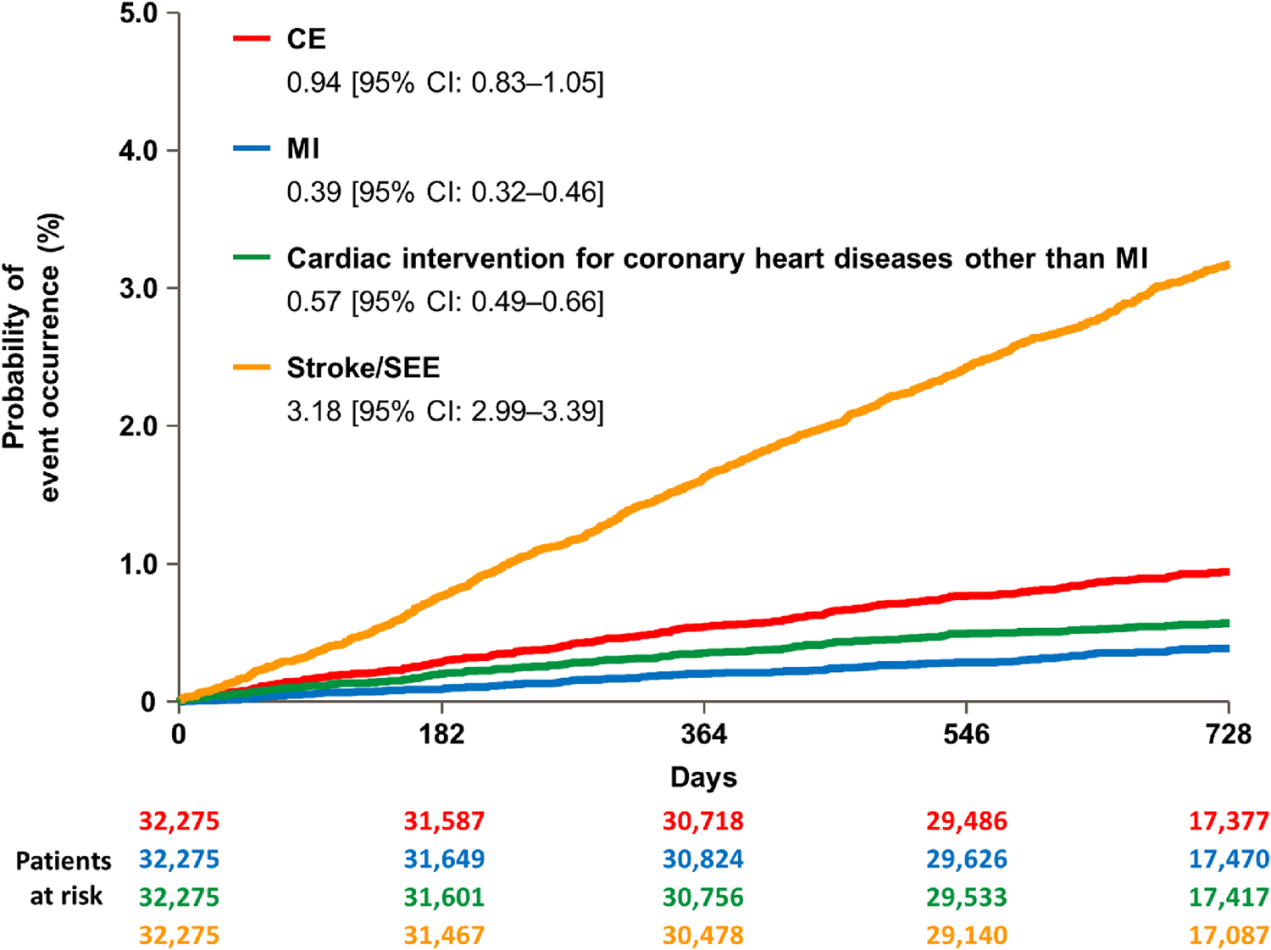
Kaplan–Meier curves for coronary events and stroke/SEE. Each occurrence and 95% CI show the data at 2 years. *CE* coronary event; *MI* myocardial infarction; *SEE* systemic embolic event

Figure 1 shows the probability of event occurrence for each clinical outcome. The probability of occurrence of CE after 2 years of observation was 0.94% [95% CI: 0.83–1.05]), which was lower than that of stroke/systemic embolic events (SEE) (3.18% [2.99–3.39]).

The incidence rate was 0.48 per 100 patient-years (95% CI: 0.42–0.53) for CE, 0.20 (0.16–0.23) for MI, and 0.29 (0.25–0.33) for that of cardiac intervention for coronary heart diseases other than MI (Table 2); these were lower than that of stroke/SEE, which was 1.62 (1.52–1.73) in the main analysis of the ANAFIE Registry [19].

**Table 2:** Incidence rates of coronary events and stroke/SEE

	CE	MI	Cardiac intervention for coronary heart diseases other than MI	Stroke/SEE^a^
Incidence, n (%)	287 (0.89)	118 (0.37)	175 (0.54)	970 (3.01)
Incidence rate (per 100 patient-years, [95% CI])	0.48 [0.42–0.53]	0.20 [0.16–0.23]	0.29 [0.25–0.33]	1.62 [1.52–1.73]

N = 32,275

*CE* coronary event; *CI* confidence interval; *MI* myocardial infarction; *SEE* systemic embolic event

^a^Data from Yamashita et al. *Eur Heart J Qual Care Clin Outcomes* 2022;8:202–213 [19]

### Risk factors of CE

The risk factors associated with new-onset CE were male sex, systolic blood pressure ≥ 130 mmHg, diabetes mellitus with glycated hemoglobin (HbA1c) ≥ 6.0%, CAD history with and without APA use, APA use without CAD history, and CrCL < 50 mL/min (Table 3). Of note, prior CAD and APA use each were significant risk factors, and their co-occurrence increased the risk even further.

**Table 3:** Risk factors for new-onset CE, MI, and cardiac intervention for coronary heart diseases other than MI during follow-up, results of a multivariate analysis

	CE				MI				Cardiac intervention for coronary heart diseases other than MI			
	N	Event (%)	HR (95% CI)	*P* value	N	Event (%)	HR (95% CI)	*P* value	N	Event (%)	HR (95% CI)	*P* value
Overall	32,275	287 (0. 9)	–		32,275	118 (0.4)	–		32,275	175 (0.5)	–	
Sex												
Male^a^	18,482	210 (1.1)	–		18,482	75 (0.4)	–		18,482	139 (0.8)	–	
Female	13,793	77 (0. 6)	0.54 (0.40–0.73)	< 0.001	13,793	43 (0.3)	0.72 (0.47–1.11)	0.136	13,793	36 (0.3)	0.43 (0.28–0.65)	< 0.001
Age, years												
< 85^a^	23,856	225 (0.9)	–		23,856	84 (0.4)	–		23,856	147 (0.6)	–	
≥ 85	8419	62 (0.7)	0.80 (0.58–1.10)	0.168	8419	34 (0.4)	0.90 (0.57–1.43)	0.664	8419	28 (0.3)	0.67 (0.43–1.05)	0.079
Body mass index (kg/m^2^)												
< 18.5	2059	11 (0.5)	0.49 (0.23–1.06)	0.069	2059	4 (0.2)	0.26 (0.06–1.06)	0.060	2059	7 (0.3)	0.72 (0.29–1.78)	0.474
> 18.5 and < 25.0^a^	17,621	167 (0.9)	–		17,621	70 (0.4)	–		17,621	101 (0.6)	–	
≥ 25.0	8090	64 (0.8)	0.89 (0.65–1.21)	0.455	8090	27 (0.3)	0.99 (0.62–1.60)	0.977	8090	39 (0.5)	0.83 (0.56–1.24)	0.368
Smoking habit												
Yes (current smoker)	1243	12 (1.0)	0.94 (0.53–1.69)	0.843	1243	5 (0.4)	1.07 (0.43–2.65)	0.885	1243	8 (0.6)	0.96 (0.47–1.96)	0.908
None (never smoked or stopped)^a^	26,068	228 (0.9)	–		26,068	97 (0.4)	–		26,068	136 (0.5)	–	
Blood pressure												
SBP < 130 mmHg^a^	16,218	125 (0.8)	–		16,218	51 (0.3)	–		16,218	77 (0.5)	–	
SBP ≥ 130 mmHg to < 140 mmHg	6937	72 (1.0)	1.59 (1.16–2.18)	0.004	6937	32 (0.5)	1.85 (1.15–2.98)	0.011	6937	42 (0.6)	1.41 (0.93–2.15)	0.107
SBP ≥ 140 mmHg	6528	65 (1.0)	1.56 (1.12–2.16)	0.008	6528	24 (0.4)	1.43 (0.85–2.42)	0.176	6528	41 (0.6)	1.57 (1.03–2.37)	0.035
Diabetes mellitus												
Presence (HbA1c < 6.0%)	1259	11 (0.9)	0.91 (0.46–1.80)	0.787	1259	5 (0.4)	1.00 (0.36–2.76)	0.993	1259	6 (0.5)	0.82 (0.33–2.04)	0.669
Presence (HbA1c ≥ 6.0%)	5824	87 (1.5)	1.48 (1.10–1.99)	0.010	5824	29 (0.5)	1.27 (0.79–2.05)	0.321	5824	61 (1.0)	1.66 (1.14–2.42)	0.008
None^a^	23,542	167 (0.7)	–		23,542	75 (0.3)	–		23,542	95 (0.4)	–	
Heart failure, left ventricular systolic dysfunction												
Yes	12,277	123 (1.00)	1.15 (0.88–1.51)	0.298	12277	50 (0.41)	0.96 (0.63–1.46)	0.855	12,277	77 (0.6)	1.37 (0.97–1.93)	0.070
None^a^	19,998	164 (0.8)	–		19,998	68 (0.3)	–		19,998	98 (0.5)	–	
Previous CAD and/or taking APA												
No CAD/no APA^a^	22,908	115 (0.5)	–		22,908	55 (0.2)	–		22,908	63 (0.3)	–	
No CAD/with APA	2653	23 (0.9)	1.81 (1.12–2.92)	0.016	2653	14 (0.5)	2.08 (1.11–3.90)	0.022	2653	10 (0.4)	1.52 (0.74–3.11)	0.251
With CAD/no APA	3663	51 (1.4)	2.50 (1.70–3.67)	< 0.001	3663	16 (0.4)	1.66 (0.88–3.10)	0.115	3663	35 (1.0)	3.11 (1.91–5.07)	< 0.001
With CAD/with APA	3051	98 (3.2)	5.76 (4.19–7.92)	< 0.001	3051	33 (1.1)	3.78 (2.30–6.22)	< 0.001	3051	67 (2.2)	7.40 (4.91–11.16)	< 0.001
Anticoagulants												
Warfarin^a^	8233	81 (1.0)	–		8233	39 (0.5)	–		8233	42 (0.5)	–	
None	2445	22 (0.9)	0.83 (0.49–1.40)	0.486	2445	11 (0.4)	1.03 (0.51–2.06)	0.937	2445	12 (0.5)	0.74 (0.34–1.61)	0.447
DOAC	21,585	184 (0.9)	1.05 (0.78–1.41)	0.747	21,585	68 (0.3)	0.86 (0.56–1.34)	0.512	21,585	121 (0.6)	1.27 (0.86–1.88)	0.234
Dyslipidemia												
Yes	13,728	156 (1.1)	1.11 (0.85–1.46)	0.432	13,728	62 (0.5)	1.23 (0.82–1.86)	0.317	13,728	98 (0.7)	1.05 (0.74–1.50)	0.780
None^a^	18,547	131 (0.7)	–		18,547	56 (0.3)	–		18,547	77 (0.4)	–	
Creatinine clearance (mL/min)												
< 30 mL/min/severe renal dysfunction/dialysis	4015	51 (1.3)	2.50 (1.64–3.82)	< 0.001	4,015	32 (0.8)	5.77 (3.02–11.02)	< 0.001	4015	19 (0.5)	1.21 (0.65–2.24)	0.542
30 mL/min to < 50 mL/min	10,685	105 (1.0)	1.57 (1.13–2.19)	0.008	10,685	40 (0.4)	2.26 (1.27–4.02)	0.006	10,685	69 (0.6)	1.35 (0.91–2.02)	0.141
≥ 50 mL/min^a^	11,561	77 (0.7)	–		11,561	23 (0.2)	–		11,561	56 (0.5)	–	

*APA* antiplatelet agent; *CAD* coronary artery disease; *CE* coronary event; *CI* confidence interval; *DOAC* direct oral anticoagulant; *HbA1c* glycated hemoglobin; *HR* hazard ratio; *MI* myocardial infarction; *SBP* systolic blood pressure

^a^Reference group

Risk factors for MI were systolic blood pressure between 130 and 140 mmHg, no history of CAD with APA and history of CAD with APA vs no history of CAD without APA, and CrCL < 50 mL/min.

Risk factors for cardiac intervention for coronary heart diseases other than MI were male sex, systolic blood pressure ≥ 140 mmHg, diabetes mellitus with HbA1c ≥ 6.0%, history of CAD/no APA, and history of CAD/APA (Table 3). Of note, OAC use was not associated with the risk of CE.

### Analysis of patient backgrounds based on the history of CAD and use of APA

Table 4 summarizes the comparison of background factors of patients with a history of CAD, with and without the use of APA. Patients using APA were significantly more likely to be male, slightly younger, and with higher BMI, lower CrCL, and higher HAS-BLED score. They were more likely to have paroxysmal AF, use OAC therapy, and had a higher proportion of comorbidities, diabetes mellitus, dyslipidemia, atherosclerotic diseases other than CAD (i.e., atherosclerotic infarction or peripheral artery disease), and chronic kidney disease.

**Table 4:** Patient characteristics according to a history of coronary artery disease and the use of antiplatelet agents

	History of CAD			No history of CAD		*P* value^d^
	With APA^a^ (n = 3051)	Without APA (n = 3663)	*P* value^b^	With APA^c^ (n = 2653)	Without APA (n = 22,908)	
Male	2215 (72.6)	2141 (58.4)	< 0.001	1640 (61.8)	12,486 (54.5)	< 0.001
Age, years	81.4 ± 4.7	81.9 ± 4.8	< 0.001	81.7 ± 4.8	81.4 ± 4.8	< 0.001
Body mass index, kg/m^2^	23.7 ± 3.5	23.4 ± 3.5	0.004	23.5 ± 3.5	23.3 ± 3.6	< 0.001
SBP, mmHg	126.0 ± 17.7	126.1 ± 16.6	0.753	127.7 ± 16.8	127.7 ± 17.0	< 0.001
DBP, mmHg	68.7 ± 11.6	69.5 ± 11.4	0.010	70.4 ± 11.7	71.1 ± 11.6	< 0.001
Creatinine clearance, mL/min	46.4 ± 16.9	46.9 ± 17.3	0.224	46.9 ± 17.6	49.0 ± 18.6	< 0.001
CHADS_2_ score	3.2 ± 1.2	3.2 ± 1.2	0.460	3.3 ± 1.3	2.7 ± 1.1	< 0.001
HAS-BLED score	2.6 ± 0.7	1.7 ± 0.8	< 0.001	2.7 ± 0.7	1.7 ± 0.8	< 0.001
History of major bleeding	141 (4.6)	195 (5.3)	0.189	128 (4.8)	975 (4.3)	0.022
AF type						
Paroxysmal	1468 (48.1)	1663 (45.4)	0.004	1090 (41.1)	9365 (40.9)	< 0.001
Persistent	744 (24.4)	1025 (28.0)	–	838 (31.6)	7,094 (31.0)	–
Permanent	839 (27.5)	975 (26.6)	–	725 (27.3)	6,449 (28.2)	–
OACs	2662 (87.3)	3429 (93.6)	< 0.001	2202 (83.0)	21,537 (94.0)	< 0.001
Warfarin	932 (30.5)	945 (25.8)	< 0.001	766 (28.9)	5590 (24.4)	< 0.001
DOACs	1729 (56.7)	2484 (67.8)	< 0.001	1434 (54.1)	15,938 (69.6)	< 0.001
History of non-pharmacological therapy for AF	516 (16.9)	830 (22.7)	< 0.001	422 (15.9)	3909 (17.1)	< 0.001
Catheter ablation	247 (8.1)	379 (10.3)	0.002	186 (7.0)	2,158 (9.4)	< 0.001
Comorbidities						
Hypertension	2520 (82.6)	2979 (81.3)	0.179	2105 (79.3)	16,708 (72.9)	< 0.001
Diabetes mellitus	1342 (44.0)	1422 (38.8)	< 0.001	805 (30.3)	5164 (22.5)	< 0.001
Dyslipidemia	2150 (70.5)	1993 (54.4)	< 0.001	1293 (48.7)	8292 (36.2)	< 0.001
Chronic kidney disease	839 (27.5)	719 (19.6)	< 0.001	602 (22.7)	4545 (19.8)	< 0.001
Myocardial infarction	1158 (38.0)	693 (18.9)	< 0.001	0 (0.0)	0 (0.0)	< 0.001
Angina	2333 (76.5)	3188 (87.0)	< 0.001	0 (0.0)	0 (0.0)	< 0.001
Heart failure, left ventricular systolic dysfunction	1322 (43.3)	1854 (50.6)	< 0.001	985 (37.1)	8116 (35.4)	< 0.001
Cerebrovascular disease	797 (26.1)	924 (25.2)	0.402	1069 (40.3)	4513 (19.7)	< 0.001
Atherosclerotic infarction	92 (3.0)	64 (1.7)	< 0.001	178 (6.7)	321 (1.4)	< 0.001
Cardiogenic infarction	163 (5.3)	202 (5.5)	0.757	268 (10.1)	1744 (7.6)	< 0.001
Lacunar infarction	174 (5.7)	183 (5.0)	0.199	260 (9.8)	819 (3.6)	< 0.001
Peripheral arterial disease^e^	476 (15.6)	398 (10.9)	< 0.001	327 (12.3)	730 (3.2)	< 0.001
Fall within 1 year	237 (7.8)	321 (8.8)	0.054	208 (7.8)	1,581 (6.9)	< 0.001

Data are presented as n (%)

*APA* antiplatelet agent; *AF* atrial fibrillation; *CAD* coronary artery disease; *DBP* diastolic blood pressure; *DOAC* direct oral anticoagulant; *OAC* oral anticoagulants; *SBP* systolic blood pressure

^a^Included 2,092 aspirin users, 849 P2Y12-inhibitor users, and 452 others (of whom 201 used dual antiplatelet therapy)

^b^Comparison between patients with a history of CAD receiving APAs and those not receiving APAs

^c^Included 1,399 aspirin users, 529 P2Y12-inhibitor users, and 819 others (of whom 39 used dual antiplatelet therapy)

^d^Comparison among the four groups

^e^Aortic plaque, internal carotid artery stenosis, and arteriosclerosis obliterans

### Bleeding events in patients with CE

**Fig. 2:**
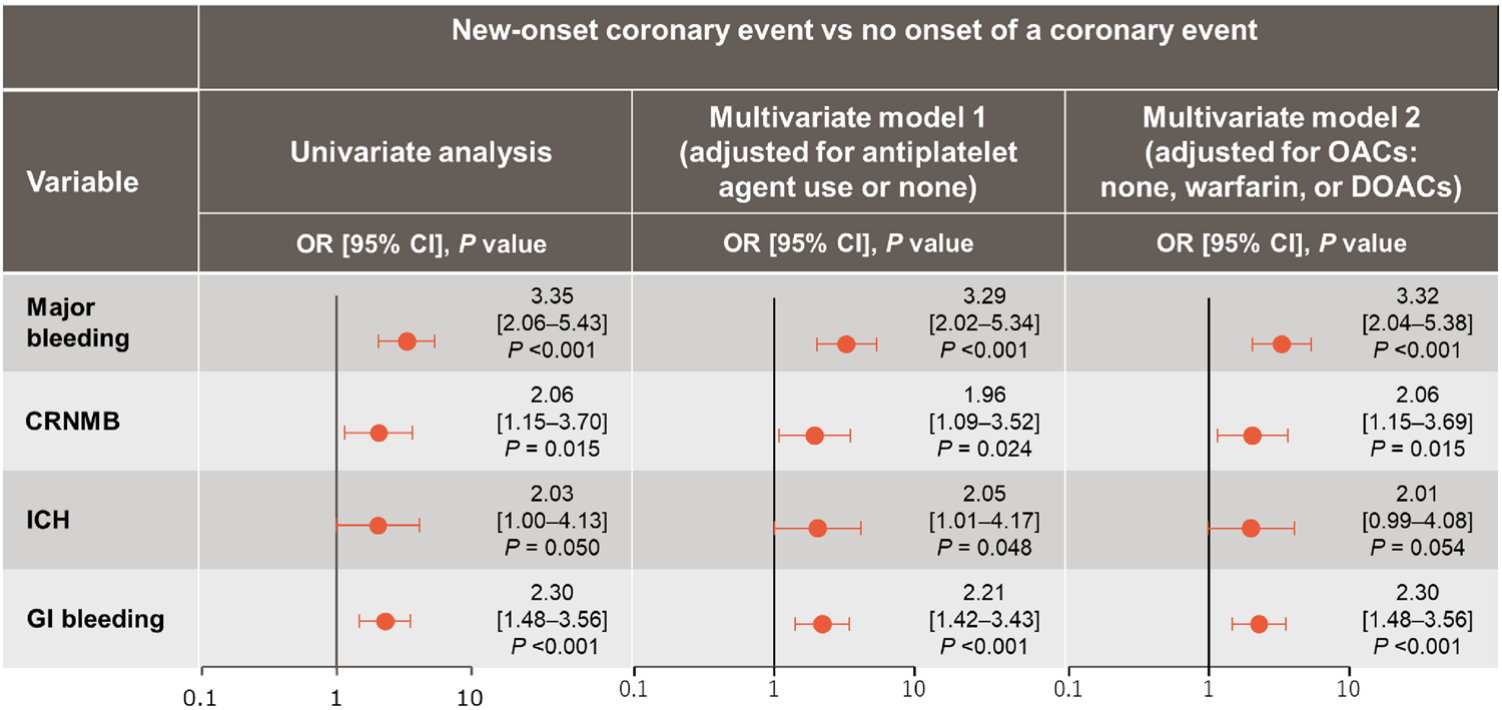
Risk of bleeding events with new-onset coronary event versus no onset of a coronary event. *CI* confidence interval; *CRNMB* clinically relevant non-major bleeding; *DOAC* direct oral anticoagulant; *GI* gastrointestinal; *ICH* intracranial hemorrhage; *OAC* oral anticoagulant; *OR* odds ratio

All bleeding events were observed in 2,557 cases (7.9%). Bleeding events in patients with or without new-onset CE are shown in Fig. 2. In the univariate analysis, new-onset CE was significantly associated with a higher incidence of major bleeding (OR: 3.35 [95% CI: 2.06–5.43]), CRNMB (2.06 [1.15–3.70]), ICH (2.03 [1.00–4.13]), and GI bleeding (2.30 [1.48–3.56]) compared with those without CE. In the multivariate analysis both when adjusting for the presence of APAs and when adjusting for the type of OACs, the incidence of major bleeding, CRNMB, ICH, and GI bleeding remained significantly higher for patients with new-onset CE compared with patients without CE. Of 287 patients who developed new-onset CE, the incidence of CE was 5.2% (n = 15) after all bleeding episodes, 2.4% (n = 7) after a major bleeding event, and 1.0% (n = 3) after a CRNMB event.

## Discussion

The main findings of this sub-analysis were as follows. First, in the overall population, the incidence rates of CE, MI, and cardiac intervention for coronary heart diseases other than MI were 0.48, 0.20, and 0.29 per 100 patient-years, respectively, which were lower than that of stroke/SEE (1.62 per 100 patient-years) reported in the main analysis of the ANAFIE Registry [19]. Second, compared with patients without CE (n = 31,988), those with new-onset CE (n = 287) were more likely to have lower CrCL and higher CHADS_2_ and HAS-BLED scores. Third, risk factors significantly associated with the onset of CE were male sex, systolic blood pressure of ≥ 130 mmHg, diabetes mellitus with HbA1c ≥ 6.0%, history of CAD, APA use, and CrCL < 50 mL/min. Fourth, the incidence of major bleeding, CRNMB, ICH, and GI bleeding was significantly higher in patients with new-onset CE compared with those without CE.

The incidence of CE in the present study was consistent with a recent report on the trends of antithrombotic therapy status and outcomes in Japanese AF patients (mean age ± standard deviation, 73.6 ± 10.9 years), in which the incidence of MI was 0.2% and that of stroke/SEE, 2.2% per patient-year during a 5-year follow-up [26]. The trends observed for the onset of CE in patients with a history of CAD were similar to those reported in a previous study overseas in which cardiovascular outcomes of patients with a history of CAD (i.e., MI) were worse than those of patients without a history of CAD [11]. However, the incidence of MI during anticoagulant therapy was lower in Japanese than in Western patients—a finding that might be attributable to ethnic differences [27–29].

The risk factors for CE for elderly patients with NVAF in the present analysis were also generally consistent with previous reports [30]. An unexpected finding of our study was that APA use was a risk factor for CE. Based on the comparison of patient background data, advanced atherosclerotic diseases are likely the basis for APA use. Therefore, patient background factors, for instance a history of atherosclerotic disease such as peripheral arterial disease, may be associated with high CE risk in patients taking APA. In these patients, it may be difficult to reduce the risk of CE, even with APA. Conversely, OACs were not associated with CE risk.

Another explanation is that bleeding associated with antiplatelet administration may have increased CE. In the present study, new-onset CE was significantly associated with a higher incidence of bleeding compared with patients without CE. The incidence of major bleeding in patients with new-onset CE was significantly higher than that in patients without CE (OR: 3.35 [95% CI: 2.06–5.43]). The findings of the multivariate analysis suggest that APA use or type of OAC at baseline may not have contributed to the incidence of bleeding in patients with new-onset CE. On the other hand, it has also been noted that bleeding complications are followed by ischemic events. In the present study, the incidence of CE after the onset of all bleeding was 5.2%, which is consistent with a previous report [13]. This may be because of the cessation of antithrombotic therapy, blood transfusion, or other interventions. As noted, adjusting for antiplatelet or anticoagulant drug use did not significantly change the OR. Therefore, these drugs do not appear to contribute to increased bleeding events in new-onset CE patients, which may instead be attributable to risk factors identified in the high bleeding risk criteria for PCI patients [31], although this remains unclear.

Concerning risk factors specifically for MI, systolic blood pressure between 130 and 140 mmHg, no history of CAD/APA, history of CAD/APA, and CrCL < 50 mL/min were significant risk factors, which is generally consistent with the previously reported risk factors for MI [32, 33]. Nevertheless, it was surprising that dyslipidemia and diabetes mellitus were not among the relevant risk factors identified. It is possible that the small sample size in that subgroup precluded these factors from reaching a significant difference. Additionally, if the administration of statins was high in this population, it is possible that the risk was modified. Nevertheless, data on statin administration were not collected in this study. Furthermore, it is possible that traditional risk factors, such as hyperlipidemia and diabetes, have become relatively unimportant in the elderly [34, 35]. Treatment history of dyslipidemia was also not recorded in this study.

Regarding the clinical relevance and implications of these findings, it is important to characterize patients at higher risk of CE to prevent and reduce CE. Our results clarify the risk of developing CE in elderly NVAF patients. Although these patients were at a lower risk of CE than stroke/SEE, strict management is required to prevent CE in patients with risk factors for CE, especially those with a history of CAD and those who require APA therapy. Another important finding was that aspirin use was a negative predictor of the development of CE. Therefore, concomitant antiplatelet therapy for primary and secondary prevention of CE may not be recommended for elderly patients with AF. These findings are consistent with the AFIRE trial [12] and are a further advance on the ASPREE trial, in which the use of low-dose aspirin in elderly patients without atrial fibrillation resulted in a significantly increased risk of major bleeding without a decreased risk of cardiovascular disease [36], although the lack of data on treatment changes after each event in the present study means that this requires further verification.

### Limitations

The main limitations of the ANAFIE Registry have been published previously [25, 37]. Information such as the withdrawal or change of APA and anticoagulants and use of statins or other treatments for dyslipidemia was not evaluated during the study. Because there was a low prevalence of a history of MI in the study population (5.7%), our findings cannot be extrapolated to populations with higher MI prevalence.

## Conclusions

This sub-analysis of the ANAFIE Registry is the first large-scale study to report that CE incidence was lower than that of stroke/SEE in elderly patients with NVAF. Risk factors for CE in elderly Japanese patients with NVAF were male sex, systolic blood pressure of ≥ 130 mmHg, diabetes mellitus, CE history, antiplatelet agent use, and CrCL of < 50 mL/min. New-onset CE was associated with a higher incidence of major bleeding than no CE. Thus, the current findings may contribute to the understanding of the management of elderly NVAF patients.

## Data Availability

The study protocol will be made available. The deidentified participant data used in this study will be shared with researchers who participated in the study and provide a methodologically sound proposal for 36 months after article publication. The proposal may be reviewed by a committee led by Daiichi Sankyo. For any purpose, requests must be in writing and should be sent to yamt-tky@umin.ac.jp. To gain access, those requesting the data will need to sign a data access agreement.

## References

[1] Wolf PA, Abbott RD, Kannel WB (1991). Atrial fibrillation as an independent risk factor for stroke: the Framingham study. Stroke.

[2] Kavousi M (2020). Differences in epidemiology and risk factors for atrial fibrillation between women and men. Front Cardiovasc Med.

[3] Heeringa J, van der Kuip DA, Hofman A, Kors JA, van Herpen G, Stricker BH (2006). Prevalence, incidence and lifetime risk of atrial fibrillation: the Rotterdam study. Eur Heart J.

[4] Zoni-Berisso M, Lercari F, Carazza T, Domenicucci S (2014). Epidemiology of atrial fibrillation: European perspective. Clin Epidemiol.

[5] Mekhael M, Marrouche N, Hajjar AHE, Donnellan E (2022). The relationship between atrial fibrillation and coronary artery disease: understanding common denominators. Trends Cardiovasc Med.

[6] Michniewicz E, Mlodawska E, Lopatowska P, Tomaszuk-Kazberuk A, Malyszko J (2018). Patients with atrial fibrillation and coronary artery disease - double trouble. Adv Med Sci.

[7] Soliman EZ, Safford MM, Muntner P, Khodneva Y, Dawood FZ, Zakai NA (2014). Atrial fibrillation and the risk of myocardial infarction. JAMA Intern Med.

[8] Aronow WS, Ahn C, Mercando AD, Epstein S (1995). Correlation of atrial fibrillation, paroxysmal supraventricular tachycardia, and sinus rhythm with incidences of new coronary events in 1359 patients, mean age 81 years, with heart disease. Am J Cardiol.

[9] Liang F, Wang Y (2021). Coronary heart disease and atrial fibrillation: a vicious cycle. Am J Physiol Heart Circ Physiol.

[10] GBD 2017 Causes of Death Collaborators (2017). Global, regional, and national age-sex-specific mortality for 282 causes of death in 195 countries and territories 1980–2017: a systematic analysis for the global burden of disease study 2017. Lancet.

[11] Mahaffey KW, Stevens SR, White HD, Nessel CC, Goodman SG, Piccini JP (2014). Ischaemic cardiac outcomes in patients with atrial fibrillation treated with vitamin K antagonism or factor Xa inhibition: results from the ROCKET AF trial. Eur Heart J.

[12] Yasuda S, Kaikita K, Akao M, Ako J, Matoba T, Nakamura M, AFIRE Investigators (2019). Antithrombotic therapy for atrial fibrillation with stable coronary disease. N Engl J Med.

[13] Kaikita K, Yasuda S, Akao M, Ako J, Matoba T, Nakamura M (2021). Bleeding and subsequent cardiovascular events and death in atrial fibrillation with stable coronary artery disease: insights from the AFIRE trial. Circ Cardiovasc Interv.

[14] Zelniker TA, Ruff CT, Wiviott SD, Blanc JJ, Cappato R, Nordio F (2019). Edoxaban in atrial fibrillation patients with established coronary artery disease: insights from ENGAGE AF-TIMI 48. Eur Heart J Acute Cardiovasc Care.

[15] Hohnloser SH, Oldgren J, Yang S, Wallentin L, Ezekowitz M, Reilly P (2012). Myocardial ischemic events in patients with atrial fibrillation treated with dabigatran or warfarin in the RE-LY (randomized evaluation of long-term anticoagulation therapy) trial. Circulation.

[16] Bahit MC, Lopes RD, Wojdyla DM, Hohnloser SH, Alexander JH, Lewis BS (2013). Apixaban in patients with atrial fibrillation and prior coronary artery disease: Insights from the ARISTOTLE trial. Int J Cardiol.

[17] Masunaga N, Ogawa H, Minami K, Ishigami K, Ikeda S, Doi K (2022). Association of concomitant coronary artery disease with cardiovascular events in patients with atrial fibrillation - the Fushimi AF registry. Circ J.

[18] Abe M, Masunaga N, Ishii M, Doi K, Ishigami K, Ikeda S (2020). Current status of percutaneous coronary intervention in patients with atrial fibrillation: the Fushimi AF registry. J Cardiol.

[19] Yamashita T, Suzuki S, Inoue H, Akao M, Atarashi H, Ikeda T (2022). Two-year outcomes of more than 30 000 elderly patients with atrial fibrillation: results from the all nippon AF in the elderly (ANAFIE) registry. Eur Heart J Qual Care Clin Outcomes.

[20] Yamashita T, Akao M, Atarashi H, Ikeda T, Koretsune Y, Okumura K (2022). Effect of polypharmacy on clinical outcomes in elderly patients with non-valvular atrial fibrillation- a sub-analysis of the ANAFIE registry. Circ J.

[21] Akishita M, Suzuki S, Inoue H, Akao M, Atarashi H, Ikeda T (2022). Frailty and outcomes in older adults with non-valvular atrial fibrillation from the ANAFIE registry. Arch Gerontol Geriatr.

[22] Hiasa KI, Kaku H, Kawahara G, Inoue H, Yamashita T, Akao M (2022). Echocardiographic structure and function in elderly patients with atrial fibrillation in Japan- The ANAFIE echocardiographic substudy. Circ J.

[23] Okumura K, Yamashita T, Akao M, Atarashi H, Ikeda T, Koretsune Y (2022). Oral anticoagulants in very elderly nonvalvular atrial fibrillation patients with high bleeding risks: ANAFIE registry. JACC Asia.

[24] Kario K, Hasebe N, Okumura K, Yamashita T, Akao M, Atarashi H (2022). Home blood pressure can predict the risk for stroke/bleeding events in elderly patients with nonvalvular atrial fibrillation from the ANAFIE registry. Hypertension.

[25] Inoue H, Yamashita T, Akao M, Atarashi H, Ikeda T, Okumura K (2018). Prospective observational study in elderly patients with non-valvular atrial fibrillation: Rationale and design of the all nippon AF in the elderly (ANAFIE) registry. J Cardiol.

[26] Akao M, Ogawa H, Masunaga N, Minami K, Ishigami K, Ikeda S (2022). 10-year trends of antithrombotic therapy status and outcomes in Japanese atrial fibrillation patients - the Fushimi AF registry. Circ J.

[27] Cho H, Kang J, Kim HS, Park KW (2020). Ethnic differences in oral antithrombotic therapy. Korean Circ J.

[28] de Hoog VC, Lim SH, Bank IE, Gijsberts CM, Ibrahim IB, Kuan WS (2016). Ethnic differences in clinical outcome of patients presenting to the emergency department with chest pain. Eur Heart J Acute Cardiovasc Care.

[29] Saito Y, Kobayashi Y, Tanabe K, Ikari Y (2020). Antithrombotic therapy after percutaneous coronary intervention from the Japanese perspective. Cardiovasc Interv Ther.

[30] Lip GY, Nieuwlaat R, Pisters R, Lane DA, Crijns HJ (2010). Refining clinical risk stratification for predicting stroke and thromboembolism in atrial fibrillation using a novel risk factor-based approach: the Euro heart survey on atrial fibrillation. Chest.

[31] Urban P, Mehran R, Colleran R, Angiolillo DJ, Byrne RA, Capodanno D (2019). Defining high bleeding risk in patients undergoing percutaneous coronary intervention: a consensus document from the academic research consortium for high bleeding risk. Eur Heart J.

[32] Brown JC, Gerhardt TE, Kwon E. Risk Factors For Coronary Artery Disease. [Updated 2023 Jan 23]. In: StatPearls [Internet]. Treasure Island (FL): StatPearls Publishing; 2023. Available from: https://www.ncbi.nlm.nih.gov/books/NBK554410/. 32119297

[33] Sanchis-Gomar F, Perez-Quilis C, Leischik R, Lucia A (2016). Epidemiology of coronary heart disease and acute coronary syndrome. Ann Transl Med.

[34] Ahmadi SF, Streja E, Zahmatkesh G, Streja D, Kashyap M, Moradi H (2015). Reverse epidemiology of traditional cardiovascular risk factors in the geriatric population. J Am Med Dir Assoc.

[35] Dalton JE, Rothberg MB, Dawson NV, Krieger NI, Zidar DA, Perzynski AT (2020). Failure of traditional risk factors to adequately predict cardiovascular events in older populations. J Am Geriatr Soc.

[36] McNeil JJ, Wolfe R, Woods RL, Tonkin AM, Donnan GA, Nelson MR (2018). Effect of aspirin on cardiovascular events and bleeding in the healthy elderly. N Engl J Med.

[37] Koretsune Y, Yamashita T, Akao M, Atarashi H, Ikeda T, Okumura K (2019). Baseline demographics and clinical characteristics in the all nippon AF in the elderly (ANAFIE) registry. Circ J.

